# Transient interdomain interactions in free USP14 shape its conformational ensemble

**DOI:** 10.1002/pro.4975

**Published:** 2024-04-08

**Authors:** Johannes Salomonsson, Björn Wallner, Linda Sjöstrand, Pádraig D'Arcy, Maria Sunnerhagen, Alexandra Ahlner

**Affiliations:** ^1^ Department of Physics, Chemistry and Biology Linköping University Linköping Sweden; ^2^ Department of Biomedical and Clinical Sciences Linköping University Linköping Sweden

**Keywords:** DUB, molecular modeling, NMR, protein dynamics, SAXS

## Abstract

The deubiquitinase (DUB) ubiquitin‐specific protease 14 (USP14) is a dual domain protein that plays a regulatory role in proteasomal degradation and has been identified as a promising therapeutic target. USP14 comprises a conserved USP domain and a ubiquitin‐like (Ubl) domain separated by a 25‐residue linker. The enzyme activity of USP14 is autoinhibited in solution, but is enhanced when bound to the proteasome, where the Ubl and USP domains of USP14 bind to the Rpn1 and Rpt1/Rpt2 units, respectively. No structure of full‐length USP14 in the absence of proteasome has yet been presented, however, earlier work has described how transient interactions between Ubl and USP domains in USP4 and USP7 regulate DUB activity. To better understand the roles of the Ubl and USP domains in USP14, we studied the Ubl domain alone and in full‐length USP14 by nuclear magnetic resonance spectroscopy and used small angle x‐ray scattering and molecular modeling to visualize the entire USP14 protein ensemble. Jointly, our results show how transient interdomain interactions between the Ubl and USP domains of USP14 predispose its conformational ensemble for proteasome binding, which may have functional implications for proteasome regulation and may be exploited in the design of future USP14 inhibitors.

## 1 INTRODUCTION

The ubiquitin‐proteasome system (UPS) is the main pathway for controlled protein degradation, with up to 80% of cellular proteins routed this way. At its simplest level, the UPS is composed of the proteasome, a cylindrical multiprotein complex that acts as the cell's molecular shredder (Bard et al., 2018; Schweitzer et al., 2016) and a ubiquitin‐conjugating system, where a series of coordinated enzymes tag damaged or unneeded proteins with the small protein ubiquitin, triggering the formation of complex ubiquitin chains linked via isopeptide bonds. A ubiquitin removal system runs in tandem within the UPS providing an additional level of regulation, mediated by a class of enzymes, known as deubiquitinases (DUBs) that counteract the signaling mediated by the ubiquitin‐conjugating system (Nijman et al., 2005). Over 100 DUBs have been identified, grouped into seven classes based on sequence homology (Lange et al., 2022). The ubiquitin‐specific protease (USP) family is the largest and most structurally diverse (Leznicki and Kulathu, 2017) where members contain a structurally conserved consensus ubiquitin‐binding catalytic domain (USP domain) and may have variable insertions and extensions that connect the USP domain to one or more domains (Reyes‐Turcu et al., 2009).

**FIGURE 1:**
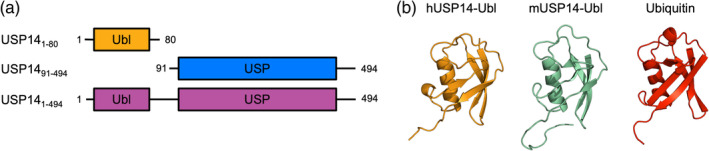
USP14 working constructs and Ubl/ubiquitin models and structures. (a) Schematic illustration of USP14 domain architecture and the constructs used in this study. (b) AlphaFold model of human USP14‐Ubl (residues 1–80, yellow), and NMR structures of murine USP14‐Ubl (residues 4–86, green, PDB‐ID 1WGG) and human ubiquitin (red, PDB‐ID 1UBQ). USP14, ubiquitin‐specific protease 14.

USP14 (Ubp6 in yeast) is one of three DUBs associated with the proteasome 19S regulatory particle (RP) and comprises an N‐terminal ubiquitin‐like (Ubl) domain and a C‐terminal USP domain, connected by a linker (Hu et al., 2005; Zhang et al., 2022) (Figure 1a). USP14 reversibly associates to the 19S RP, where the USP14 Ubl domain (USP14‐Ubl) binding site on the 19S RP nonATPase 1 (Rpn1) in yeast has been mapped by hydrogen exchange mass spectroscopy (Shi et al., 2016). This together with cryo‐EM data of the USP14‐USP domain binding site at the 19S RP ATPase 1 and ‐2 (Rpt1 and ‐2) (Huang et al., 2016), suggests a model where USP14 adopts an extended conformation where the Ubl and USP domains are bound at distinct sites on the proteasome. Recent cryo‐EM characterization of USP14‐bound proteasome suggest that a conformational continuum of states is involved in the functional cycle of the USP14 regulated proteasome, where USP14‐Ubl binding to Rpn1 plays a key role (Zhang et al., 2022), and DUB activity of USP14 is increased when bound to the proteasome (Lee et al., 2010). Emerging studies suggest that USP14 also plays an important proteasome independent role. Free USP14 is present in the cell and interacts with proteins in other systems where the Ubl domain may be vital for binding (Doherty et al., 2022; Kuo and Goldberg, 2017; Ming et al., 2022; Srinivasan et al., 2019). USP14 has emerged as a promising target for cancer and neurodegenerative conditions. Beyond its role in proteasomal activity, it influences key proteins in the cell cycle, autophagy, and canonical Wnt signaling pathways (Jung et al., 2013; Kim et al., 2018; Liu et al., 2022; Song et al., 2017). Inhibition of USP14, via pharmacological means or RNA interference has been shown to reduce cellular proliferation in both prostate and breast cancer cell lines (Liao et al., 2017; Liao et al., 2018). Recently, the USP14‐Ubl domain was found to be critical for USP14 interaction with the IDO1 protein in colorectal cancer (Shi et al., 2022).

Ubl domains are frequently found in multimodular DUBs within the USP family (Zhu et al., 2007), where they hold functions related to substrate recognition and regulation of USP enzymatic activity (Clerici et al., 2014; Faesen et al., 2011; Wolberger, 2014). USP7 comprises five Ubl domains where interactions between the USP and the Ubl 4–5 region increase ubiquitin affinity and catalytic activity (Faesen et al., 2011; Kim et al., 2019; Rougé et al., 2016), and the activation of USP4 is dependent on binding of its DUSP (domain in USP)—Ubl domain to its catalytic domain (Clerici et al., 2014). Ubl domains are not exclusive to DUBs as they are also prevalent in other types of ubiquitin binding proteins where they can regulate activity. For example, in the E3 ligase Parkin, the Ubl domain interacts with RING1 domain resulting in autoinhibition (Gladkova et al., 2018; Sauvé et al., 2015). Several Ubl domain containing proteins can also mediate proteolytic activity of the proteasome via transient interaction with the 19S RP (Collins and Goldberg, 2020; Yu et al., 2016).

It is not known whether Ubl and USP domains in USP14 interact in solution. Structures of the USP14‐USP domain alone have been solved by x‐ray crystallography, both in presence and absence of ubiquitin, and with active‐site inhibitors (Hu et al., 2005; Lin et al., 2020; Wang et al., 2018), but no structural studies of full‐length USP14 in the absence of proteasome have yet been presented. The structure of the Ubl domain in murine USP14 has been solved by nuclear magnetic resonance (NMR) spectroscopy (Zhao et al., 2004) and closely resembles that of ubiquitin (Figure 1b). Previous NMR studies have revealed how the small, stable ubiquitin entity employs conformational selection dynamics at distinct, flexible loops in order to allow specific binding to a wide range of proteins (Lange et al., 2008), and a pincer‐like mode identified by principal component analysis has been suggested to dynamically govern all ubiquitin binding events (Fenwick et al., 2011; Smith et al., 2016). Whether Ubls comprise such functionally related internal dynamics is not known.

To increase our understanding of the structural and dynamic properties of the USP14‐Ubl domain alone and in the context of full‐length USP14 in solution, we have used NMR spectroscopy and small‐angle x‐ray scattering (SAXS) in combination with molecular modeling. We find that while the Ubl domain retains a high degree of mobility with respect to the USP domain, detailed evaluation of NMR chemical shift changes, ^15^N relaxation experiments, and a conformational ensemble of full‐length USP14 obtained by SAXS indicate that the Ubl domain interacts transiently with the USP domain and/or the interdomain linker. In the conformational ensemble of USP14, we find that the relative position of the Ubl toward the USP domain is not uniformly distributed but is partly biased toward its proteasome‐bound structure (Zhang et al., 2022). We propose that this may have functional implications for proteasome regulation.

## 2 RESULTS

### 2.1 NMR assignment of USP14‐Ubl alone and in the context of full‐length USP14

**FIGURE 2:**
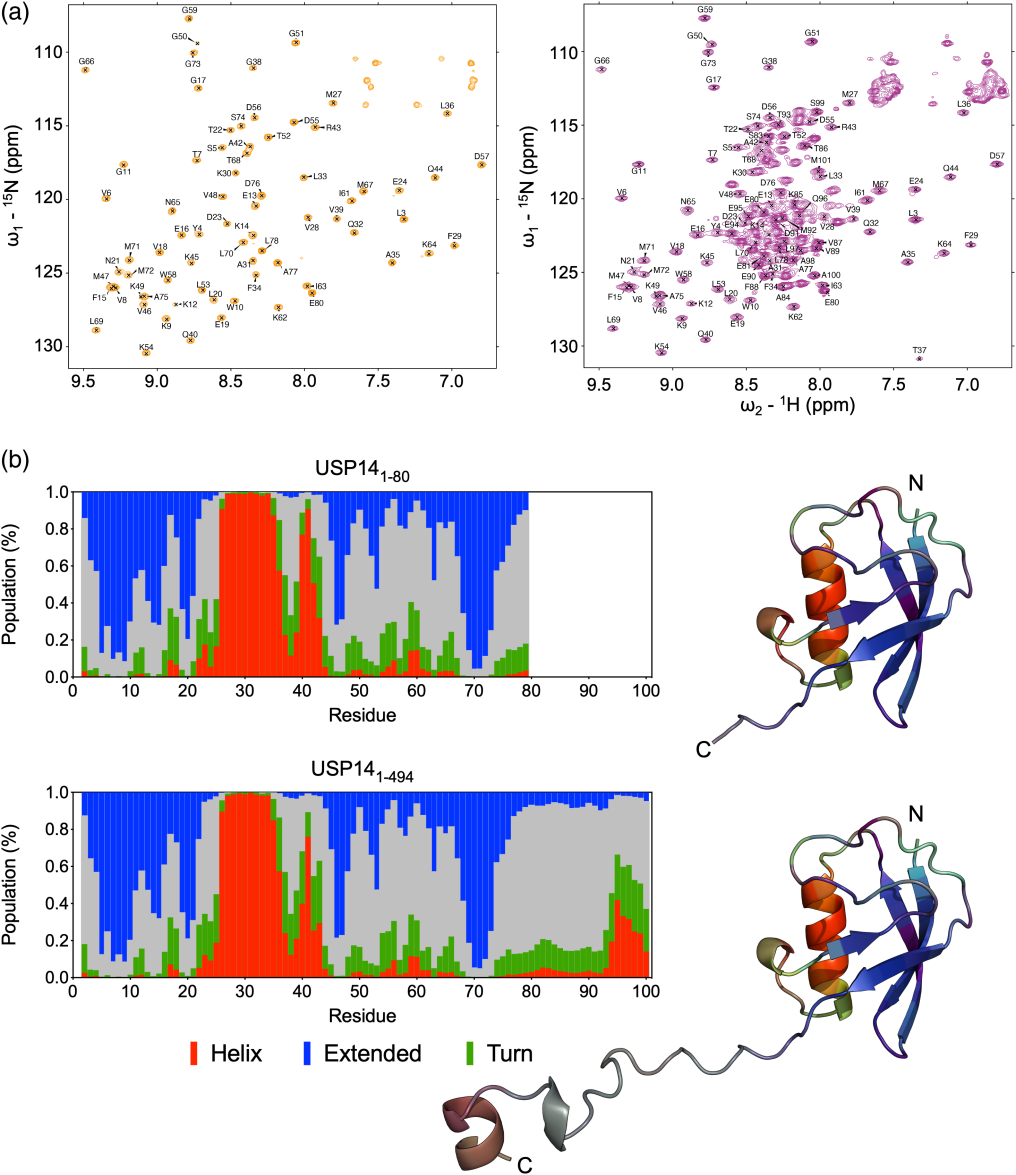
NMR assignment and secondary structure assessment of the USP14 Ubl domain alone and in the context of full‐length USP14. (a) HSQC spectra of USP14_1–80_ (yellow) and USP14_1–494_ (purple) with resonances assigned to their respective residue. (b) Secondary structure populations of the Ubl domain and linker derived by CheSPI for human USP14_1–80_ and USP14_1–494_, and their corresponding AlphaFold models colored according to their respective CheSPI plots. NMR, nuclear magnetic resonance; USP14, ubiquitin‐specific protease 14.

In this study, we investigated three constructs of human USP14 by NMR and SAXS (Figure 1a). We first employed NMR to analyze the free USP14‐Ubl domain (USP14_1–80_) and in the context of full‐length USP14 (USP14_1–494_). Near‐complete ^1^H, ^13^C, ^15^N backbone chemical shift assignments of the Ubl domain were obtained for USP14_1–80_ and USP14_1–494_. The Ubl‐corresponding peaks in USP14_1–80_ were highly resolved and well dispersed, in agreement with a well‐folded Ubl structure (Figure 2a). The USP14_1–494_ spectrum comprises a full set of Ubl resonances that correspond to those in the unattached Ubl domain in USP14_1–80_, suggesting retained structural independence of the Ubl domain within the full‐length protein. A set of high‐intensity peaks in USP14_1–494_ were assigned to the USP14 linker region (residues 76–101) connecting the Ubl and USP domains, whereas resonances in the USP domain were not observable, likely due to a much slower tumbling rate of the USP14‐USP domain (46 kDa) (Figure 2a). Secondary structure propensities predicted from chemical shifts using CheSPI (Nielsen and Mulder, 2021) suggest that the N‐terminal part of the linker region (residues 76–93) remains flexible in the full‐length construct (CheZOD *Z*‐score < 8) but may adopt transient α‐helical structure in its C‐terminal part in the intact protein (averaged *Z*‐score of 7.8 for residues 94–100) (Figure 2b), as also suggested from the AlphaFold structural model of human USP14 (Jumper et al., 2021) (AF‐P54578‐F1) (Figure 2b).

Murine USP14‐Ubl differs from human USP14‐Ubl by only two amino acids (Met63Ile, Val69Leu) (Jung et al., 2013). Its NMR structure (PDB‐ID 1WGG) agrees well with the AlphaFold structural model (Jumper et al., 2021) of the Ubl domain in human USP14 (AF‐P54578‐F1, backbone RMSD 0.78; Figure 1b), as do secondary structure propensities predicted from chemical shifts by CheSPI (Nielsen and Mulder, 2021) (Figure S1). Given the consistency of the AlphaFold model of human USP14 with structural properties observed by NMR for the Ubl domain (Figure 1b) as well as the interdomain linker (Figure 2b), and as shown later in this work also with the USP structural envelope as determined by SAXS (Figure 5), we here choose this model to visualize NMR results from USP14_1–80_ and USP14_1–494_.

**FIGURE 3:**
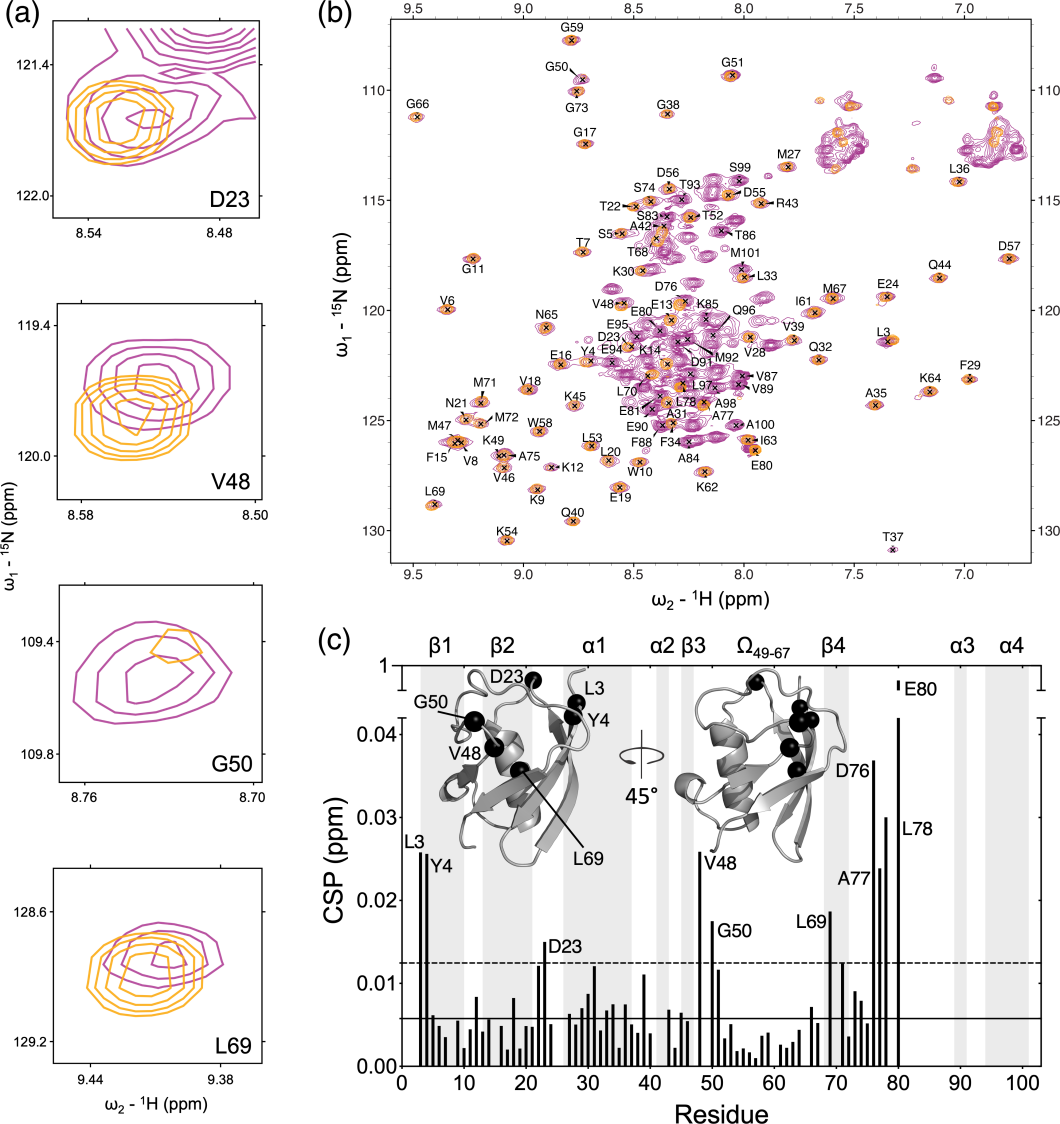
Comparison of chemical shifts for the Ubl domain in USP14_1–80_ and USP14_1–494_. (a) Selection of NMR resonances from panel (b) in the USP14‐Ubl domain displaying significant CSPs between USP14_1–80_ (yellow) and USP14_1–494_ (purple). (b) HSQC spectra of USP14_1–80_ superimposed onto USP14_1–494_. (c) CSP values comparing the Ubl domain in USP14_1–80_ and in the context of USP14_1–494_. The solid line represents the trimmed mean, with corresponding standard deviation (2*σ*) as dashed line (see Section 4 for details). CSPs larger than 2*σ* from the trimmed mean are considered significant and are shown as black spheres on the USP14‐Ubl AlphaFold model. CSPs, chemical shift perturbations; NMR, nuclear magnetic resonance; USP14, ubiquitin‐specific protease 14.

**FIGURE 4:**
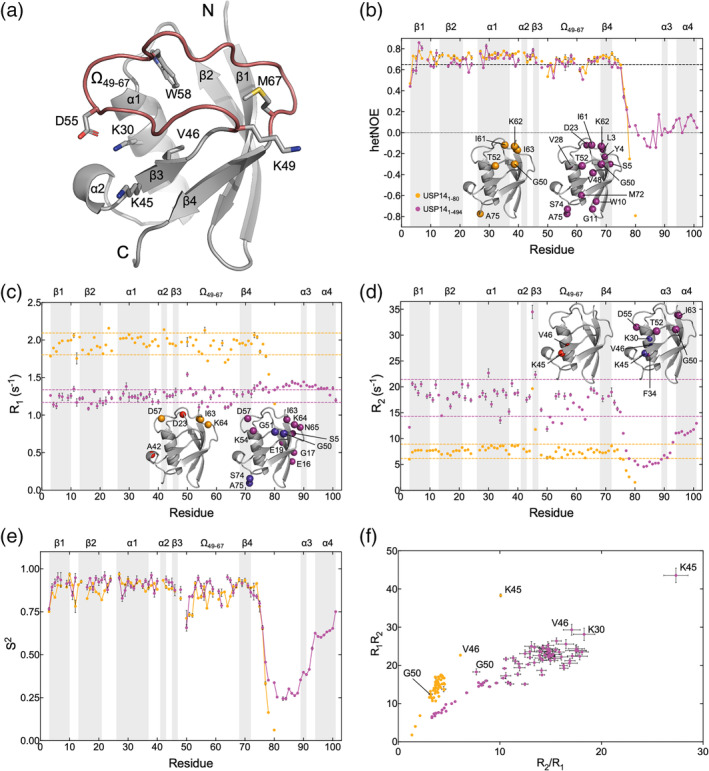
NMR relaxation evaluation of the USP14‐Ubl domain alone and in the context of full‐length USP14. (a) AlphaFold model of USP14‐Ubl (residues 1–75). Ω_49–67_‐loop highlighted in copper red. (b) HetNOE values of USP14_1–80_ (yellow) and USP14_1–494_ (purple). Dashed line indicates the value 0.65 which was used as cutoff for *τ*_c_ calculations. Residues below 0.65 for the respective USP14 constructs are shown as spheres. (c and d) *R*_1_ and *R*_2_ relaxations rates colored as in (b). Dashed lines represent 2*σ* from mean using a standard deviation‐based trimming process (see Section 4 for details). Residues above 2*σ* are indicated as blue (USP14_1–494_) or red (USP14_1–80_) spheres, and residues below 2*σ* as purple or yellow. (e) *S* ^2^ values estimated from TENSOR2. (f) Per‐residue plot of *R*_1_ *R*_2_ as a function of *R*_2_/*R*_1_, with annotated residues suggested to show motions in slower timescales in one or both contexts, based on elevated values of the *R*_1_ *R*_2_ product (Kneller et al., 2002). hetNOE, {^1^H}–^15^N Overhauser effect; NMR, nuclear magnetic resonance; USP14, ubiquitin‐specific protease 14.

Small but significant chemical shift perturbations (CSPs) were observed when comparing the unattached Ubl domain in USP14_1–80_ and the Ubl domain in the context of USP14_1–494_ (Figure 3). We noted small CSPs for residues at the N‐ and C‐termini of a surface‐lining omega‐type loop in the Ubl domain (Leszczynski and Rose, 1986; Papaleo et al., 2016) comprising residues 49–67 between strands β3 and β4, whereas the middle part of the omega loop, anchored into the domain core by W58, seems unaffected (Figures 3c and 4a). We will continue to refer to this highly conserved region in USP14 as Ω_49–67_. Together with CSPs close to the N‐terminus, these perturbations co‐localize at the same face of the USP14‐Ubl domain structure (Figure 3c). While CSPs observed in the USP14_1–80_ C‐terminus are most likely a consequence of the disconnection from the longer USP14_1–494_ context, the CSPs within the Ubl domain when comparing its free and USP‐linked state might potentially reflect dynamic, transient interactions between USP14‐Ubl and ‐USP domains.

### 2.2 NMR relaxation experiments indicate altered dynamics of Ubl free and in context of USP14

To assess whether fast dynamics (ps‐ns timescale) in the linker and Ubl regions of USP14 is affected by the presence or absence of the USP domain, steady‐state heteronuclear {^1^H}–^15^N Overhauser effect (hetNOE) and the spin–lattice (*R*_1_) and spin–spin (*R*_2_) relaxation rates were obtained for each residue, and from these data, order parameters (*S* ^2^) were calculated (Figure 4b–f). A structurally and dynamically stable Ubl domain is indicated by the high *S* ^2^s obtained, both alone and in the context of USP14. In contrast, residues in the linker region of USP14_1–494_ have hetNOE values lower than 0.4 which indicates high backbone flexibility. Slightly higher *R*_1_ and lower *R*_2_ in this region is consistent with high flexibility. According to the *S* ^2^ the linker region appears more ordered in the C‐terminal part of the linker (residue 93–101) compared to the N‐terminal part (residue 76–92) (Figure 4e). This agrees with the CheSPI analysis and the AlphaFold prediction model of USP14 where the C‐terminal part of the linker shows partial α‐helical properties (Figure 2b).

We observed variations in ps‐ns dynamics within the Ubl domain and significant but discreet differences when comparing USP14_1–80_ and USP14_1–494_ (Figure 4b–f). The Ubl domain alone overall shows uniform hetNOEs above 0.65 as expected for a stably folded structure, with lower hetNOEs at C/N‐terminal residues of the construct as expected. In agreement with the CSP pattern for Ω_49–67_, G50, T52, I61, and K62 show slightly lower‐than‐average hetNOEs in both USP14_1–80_ and USP14_1–494_, indicating increased flexibility of the omega loop terminal ends compared to the Ubl core (Figure 4b). N60 could not be assigned, possibly due to severe line broadening by chemical exchange since no unassigned peaks remained in the USP14_1–80_ spectrum. HetNOEs in the β1‐β2 loop (W10 and G11) are lower in USP14_1–494_ compared to USP14_1–80_.

The *R*_1_ relaxation rate constants also show significant but small variations within the Ubl domain of the two constructs (Figure 4c). In USP14_1–80_, residues D57, I63, and K64 within Ω_49–67_ show significantly lower *R*_1_ values compared to the rest of the domain, as well as residues in the C‐terminus as commonly observed in proteins (Kay et al., 1989). Residue D23 in the loop connecting β2 with α1 and A42 in α2 instead show significantly higher *R*_1_ values. The same residues that show lower *R*_1_ values in Ω_49–67_ for USP14_1–80_ persist in USP14_1–494_, with additionally K54 and N65. Further, E16 and E19 in β2 are also affected in USP14_1–494_, with slightly decreased *R*_1_ values. G50 and G51 instead show significantly higher *R*_1_. Interestingly, all residues displaying significantly lower *R*_1_ values in USP14_1–494_, indicating decreased backbone flexibility, are in spatial proximity within β1, β2, and Ω_49–67_ (Figure 4c).

Examining the *R*_2_ relaxation rates (Figure 4d), residues K45 and V46 located in β3 adjacent to Ω_49–67_ stand out with highly increased values in both USP14_1–80_ and USP14_1–494_. By plotting *R*_1_ *R*_2_ as a function of *R*_2_/*R*_1_ it is possible to discern residues that undergo chemical exchange as elevated values of the *R*_1_ *R*_2_ product are associated with motions in slower timescales (Kneller et al., 2002). In this representation of corresponding *R*_1_ and *R*_2_ values, K45 and V46 are clear outliers, suggesting that they undergo chemical exchange in the μs‐ms time scale (Figure 4f) (Kneller et al., 2002). In USP14_1–494_, *R*_2_ values are significantly reduced at distinct positions in Ω_49–67_ (G50, T52, D55, I63) suggesting increased flexibility in the full‐length protein. The resonance intensities of G50 as well as the heavily overlapped K49 are attenuated in both USP14_1–80_ and USP14_1–494_ but more so in USP14_1–80_ (Figure 3a). The low intensity and small linewidth of G50 could potentially be due to additional conformations giving rise to resonance frequencies that are line broadened beyond detection with conversion rates that are slow on the NMR scale. The relatively weaker signal of G50 in USP14_1–80_ suggests that the state corresponding to the detected resonance is populated to a lesser degree in USP14_1–80_, compared to G50 in USP14_1–494_. The loop corresponding to Ω_49–67_ in ubiquitin is stabilized by a conserved salt bridge between K27 and D52 (Nandi and Ainavarapu, 2022; Walters et al., 2004), homologous to K30 and D55 in USP14. The deviating *R*_2_ values of K30 and D55 in USP14_1–494_ (Figure 4d) and the distinctly high *R*_1_ *R*_2_ for K30 (Figure 4f) could potentially be due to occasional disruption of a similar salt bridge, which would agree with increased flexibility within Ω_49–67_ in the context of full‐length USP14. The modulated dynamics in and adjacent to Ω_49–67_ in USP14_1–494_ is not observed in USP14_1–80_ where, excluding K45, V46, and the flexible N/C regions, all residues show *R*_2_ values within 2*σ* from the Ubl mean.

Taken together, the USP14‐Ubl domain dynamics within β1, β2, and Ω_49–67_ is modulated in the context of USP14_1–494_ which is supported by small but significant CSPs in the same regions (Figures 3c and 4). Furthermore, residues in and adjacent to the USP14‐Ubl Ω_49–67_ loop show evidence of chemical exchange and increased flexibility in the full‐length protein. Weak or transient interdomain interactions may well induce such changes in the internal dynamics of the Ubl domain as previously noted for ubiquitin (Fenwick et al., 2011; Lange et al., 2008; Smith et al., 2016).

### 2.3 Tumbling of the Ubl domain is restricted by the USP domain

To further investigate the possible presence of a transient interaction between the Ubl and USP domains in USP14, we evaluated the tumbling rates of USP14_1–80_ and USP14_1–494_ by NMR (Table 1). The rotational correlation time (*τ*_c_) is directly correlated to the molecular weight for particles with comparable shape (Cavanagh et al., 2006). To estimate the overall tumbling rates of the Ubl domain in USP14_1–80_ and USP14_1–494_, their respective *τ*_c_ were calculated from *R*_1_ and *R*_2_ values in TENSOR2 (Table 1), after excluding the highly flexible residues (hetNOE values below 0.65) (Figure 4b). Theoretical estimates of *τ*_c_ for free and USP‐bound Ubl domain were evaluated by HYDRONMR (Huertas and Carrasco, 2000) using the AlphaFold model of the free Ubl domain and a crystal structure of USP14‐USP complex with ubiquitin (Hu et al., 2005). Previous studies suggest that domains separated by a disordered linker of 12–20 residues should see an increase in *τ*_c_ by 35%–40% (Bae et al., 2009; Walsh et al., 2010). The linker in USP14 is 25 residues long and highly flexible as judged both from chemical shifts and relaxation rates (Figures 2b and 4). However, the *τ*_c_ of the Ubl domain in full‐length USP14 increases by 119% compared to free Ubl, which is significantly higher than expected for a flexible connection, but much smaller than expected for a tight complex (Table 1). This suggests that the tumbling of the Ubl domain within USP14 is indeed more restricted than expected for a flexible connection, supporting the notion of transient interdomain interactions between Ubl and USP domains. To investigate whether these interdomain interactions affect DUB activity, we measured ubiquitin‐rhodamine hydrolysis for USP14_1–494_ and USP14_99–494_. No significant difference was observed, indicating no major catalytic regulatory role of the Ubl domain in solution (Figure S2).

**TABLE 1:** Measured and theoretical *τ*_c_ values for the Ubl domain in USP14_1–80_ and USP14_1–494_.

Measured	*τ*_c_ (ns)
Ubl in USP14_1–80_	5.4
Ubl in USP14_1–494_	11.8
Theoretical	
Free Ubl^a^	5.24
Tight complex^b^	31.7
Flexibly linked^c^	7.3, 7.5

^a^ Estimated by HYDRONMR using AlphaFold model of the USP14 Ubl domain (residues 1–75).

^b^ Estimated by HYDRONMR from crystal structure of Ub‐bound USP14‐USP (PBD‐ID 2AYO).

^c^ Estimated as described in Bae et al. (2009) and Walsh et al. (2010), respectively.

### 2.4 A conformational ensemble is required to represent full‐length USP14

**FIGURE 5:**
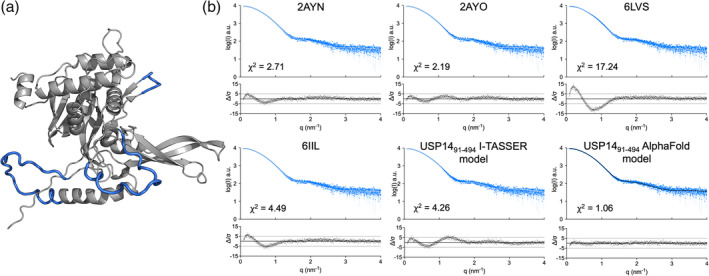
USP14‐USP structures and models compared to SAXS data. (a) AlphaFold model of USP14‐USP (residues 91–494, from AF‐P54578‐F1) with loops lacking electron densities in crystal structures highlighted in blue. (b) CRYSOL fits of USP14‐USP models and crystal structures to experimental SAXS data. Bound ligands and waters were removed prior to fit. Chain A was used for structures containing several chains. SAXS, small‐angle x‐ray scattering.

To further investigate the dynamic relation between the USP14 Ubl and USP domains, we analyzed USP14_1–494_ and USP14_91–494_ (Figure 1a) by size exclusion chromatography small‐angle x‐ray scattering (SEC‐SAXS) (Table 2). Using CRYSOL (Svergun et al., 1995), the SAXS scattering profile of USP14_91–494_ was compared to theoretical SAXS profiles of previously published crystal structures of the USP14‐USP domain which resulted in *χ* ^2^s in the range of 2.19–17.92 (Figure 5, Table S1). Since the large *χ* ^2^ is likely due to lack of electron density in loops such as 214–241 and 354–417 in the crystal structures (Figure 5a), models of the entire USP domain including these loops were generated by both I‐TASSER (Yang and Zhang, 2015) and AlphaFold (Jumper et al., 2021). The AlphaFold model showed a considerably better fit to the SAXS data, likely due to the loops being modeled as more extended from the core fold (Figure 5, Table S1), and was therefore chosen to represent the USP domain structure in subsequent atomistic modeling. SAXS Guinier and P(r) analysis jointly indicate a larger (23%) radius of gyration (*R*_g_) for USP14_1–494_ compared to USP14_91–494_, as well as a 45% increase in *D*_max_ for the full‐length protein (Table 2, Figure S3). In the dimensionless Kratky plot, USP14_91–494_ has a bell‐shaped curve with its maximum at (1.732 (√3), 1.104) (Figure S3D), which is characteristic for globular proteins (Durand et al., 2010). USP14_1–494_ has a similarly shaped curve but shows a maximum at higher values, which is indicative of a more rod‐like structure or semi‐flexibility between domains (Durand et al., 2010; Vela and Svergun, 2020). Taken together, the Kratky plot and the differences in *R*_g_ and *D*_max_ imply that USP14_1–494_ is not as globular as USP14_91–494_ and that the Ubl domain is not binding tightly to the USP domain, which agrees with the NMR data.

**TABLE 2:** SEC‐SAXS parameter summary for USP14_1–494_ and USP14_91–494_.

Instrument parameters		
Protein sample	USP14_1–494_	USP14_91–494_
Column type	S75 Increase 10/300	S75 Increase 10/300
Flow rate (mL/min)	0.6	0.6
Injection volume (μL)	75	75
Load concentration (mg/ml)	12	16
Temperature (°C)	20	20
Buffer	20 mM HEPES, pH 7.5, 150 mM NaCl, 2 mM TCEP	20 mM HEPES, pH 7.5, 150 mM NaCl, 2 mM TCEP
Sample to detector distance (m)	3	3
Exposure time/number of frames	1 s/2400	1 s/2100
X‐ray wavelength (nm)	0.124	0.124
Information content		
Intensity units	Arbitrary	Arbitrary
#Shannon channels	29	24
Highest useable *s*_max_ (nm^−1^)	7.25	7.32
Predicted *D*_*max*_ (nm)	12.56	10.22
Guinier analysis		
Guinier *I*(0) (*σ*)	1064 (0.97)	9472 (6.32)
*R*_g_ (Guinier, nm) (*σ*)	3.14 (0.005)	2.56 (0.003)
*sR*_g_ range/(points used)	0.27–1.3 (26; 144)	0.32–1.3(39; 170)
P(r) analysis		
*I*(0), POR (*σ*)	1072 (1.16)	9473 (6.53)
*R*_g_ (POR, nm) (*σ*)	3.26 (0.008)	2.56 (0.003)
*D*_max_ (nm)	12.50	8.25
Porod volume (nm^3^)	111	88
MW analysis		
Calculated MW, from amino acid sequence (kDa)	56.07	46.16
MW (Bayes, kDa)	55	46
MW credibility interval (kDa)	51–57	43–48
MW SAXSMow (kDa)	55	46
MW Vc (kDa)	55	46
Shape classification and hydrodynamics		
Ambimeter score (${\mathrm{sR}}_{g}^{\max}$)	0.4771	1.079
#shape topologies	3	12
Uniqueness	Potentially unique	Potentially unique

Abbreviation: SEC‐SAXS, size exclusion chromatography‐small‐angle x‐ray scattering.

**FIGURE 6:**
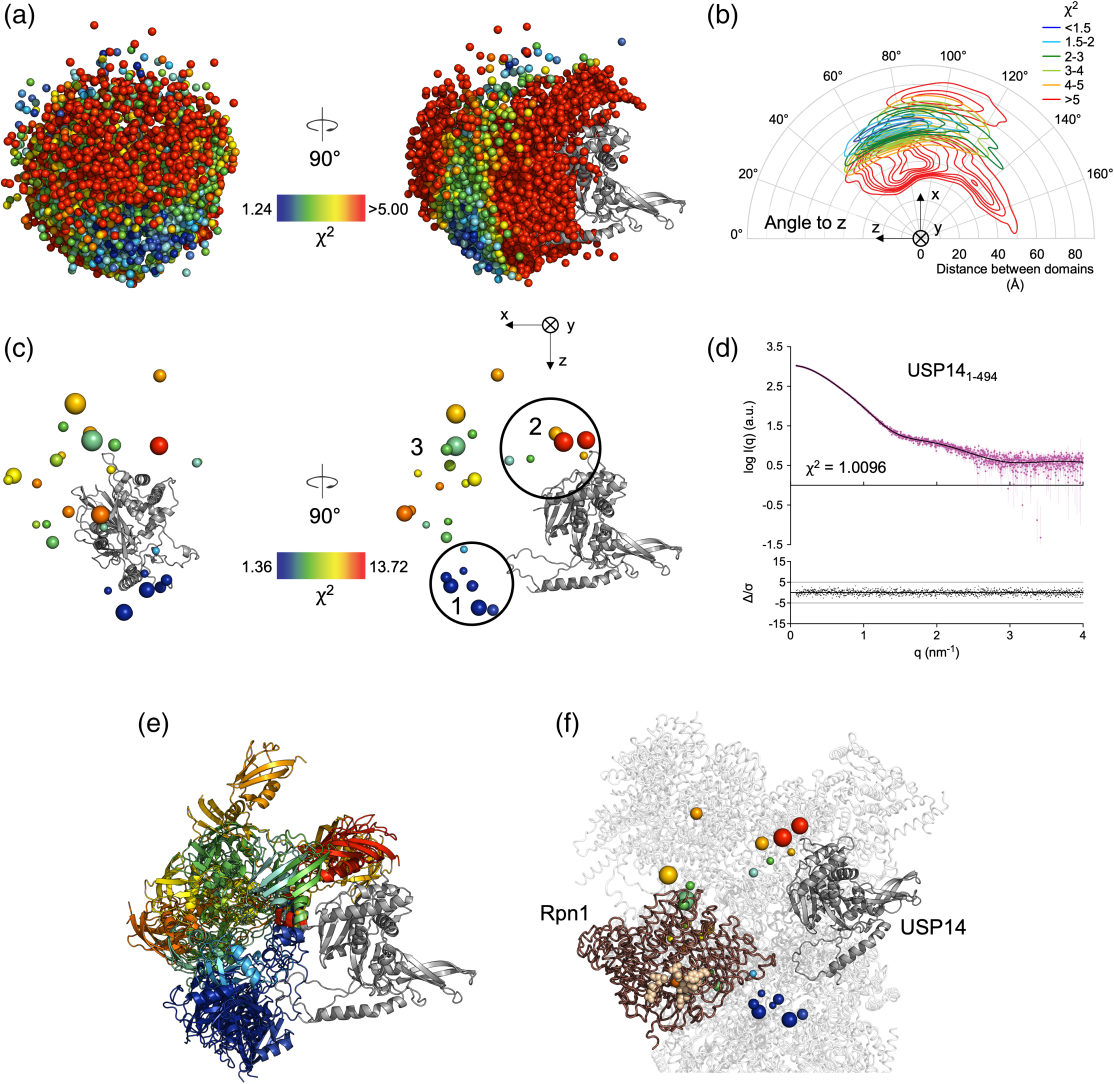
SAXS‐based molecular modeling describes the conformational ensemble of full‐length USP14. (a) Starting set of 13,000 generated models of full‐length USP14 superimposed on the USP domain, where spheres represent the center of mass for each Ubl domain. Sphere colors indicate how well each model fits USP14_1–494_ SAXS data, with red for *χ* ^2^ > 5. (b) Projected contour plot of *χ* ^2^ for all models in the starting set as a function of distance and positioning between the center of mass for the Ubl and USP domains, with coordinate system as in (a) and the USP domain center of mass in origo. (c) Conformational SAXS‐based ensemble of 30 iBME‐selected USP14 models represented as center‐of‐mass spheres as in (a), and superimposed on the USP domain. The sphere volume indicates the model weight in the ensemble and the sphere color how well each individual model fits USP14_1–494_ SAXS data, according to the rainbow bar. Models are categorized in three groups depending on the Ubl domain position relative the USP domain and have distinct *R*_g_s (see Figure S4). (d) Weighted fit of the USP14 ensemble to USP14_1–494_ SAXS data. (e) The USP14 SAXS‐based ensemble from (c) illustrated in full cartoon, structurally aligned onto the USP domain, and with Ubl domains colored as in (c). (f) USP14 conformational ensemble as in (c), aligned to the USP14‐USP domain in a cryo‐EM structure of the proteasome (PDB‐ID 7W3H). The Ubl interaction site in Rpn1 is indicated by wheat‐colored spheres. iBME, iterative Bayesian/Maximum Entropy; SAXS, small‐angle x‐ray scattering; USP14, ubiquitin‐specific protease 14.

To obtain a conformational ensemble describing the solution structure of USP14_1–494_, we used the recent iterative Bayesian/Maximum Entropy approach (iBME), which is specifically tailored to intrinsically disordered proteins and/or proteins containing domains connected by flexible linkers (Pesce and Lindorff‐Larsen, 2021). We started from a set of 13,000 widely sampled models with highly varied *χ* ^2^ fits to the SAXS data (Figure 6a, see Section 4 for details). While the position of the Ubl domain is not evenly distributed around the USP domain in this set of models probably due to restrictions imposed by the linker length, improved *χ* ^2^s are observed for certain Ubl‐USP relative positions (Figure 6b). By iBME, we then selected a USP14 structural ensemble comprising 30 models from the initial 13,000 models (Figure 6c, see Section 4 for details). This USP14 solution structural ensemble fits experimental data with a *χ* ^2^ = 1.0096, whereas the individual models in the ensemble have *χ* ^2^'s in the range of 1.36–13.72, indicating that the USP14 structural ensemble explains the SAXS data better than any single model (Figure 6d).

Members of the USP14 solution structure ensemble may be categorized into three subgroups according to the relative positioning of the Ubl domain to the USP domain (Figure 6c), which agrees well with distinct clusters of *R*_g_ as a function of *χ* ^2^ (Figure S4). In Group 1, the Ubl domain is positioned close to the USP loop including residues 385–416 and comprises models with best *χ* ^2^ fits to the SAXS data (blue in Figure 6a). Notably, the USP14_385–416_ loop is not visible in any USP14 crystal structure (Hu et al., 2005; Wang et al., 2018) but shows slight density in a recent cryo‐EM structure with USP14 bound to the proteasome, where it is involved in proteasome binding (Zhang et al., 2022). In Group 2, the Ubl domains are positioned close to two α‐helices above the USP domain catalytic site, where allosteric regulation and interactions has previously been observed in related USPs (Li et al., 2016; Samara et al., 2012). Remaining models in Group 3 extend away from the USP domain. Judged by the sum of normalized weights of the models, Groups 1 and 2, respectively represent 19.2% and 25.7% of the ensemble, while remaining extended models in Group 3 represent a majority (55.1%) of the ensemble. The small size and near‐spherical properties of the Ubl domain together with the limited spatial resolution of the SAXS experiment prevented observations of any preferred rotational orientation of the Ubl domains within any of the subgroups (Figure 6e).

### 2.5 Integrated analysis suggests that transient contacts shape the USP14 conformational ensemble

Using an integrative structural biology approach, we probed whether a unified model would be able to integrate all the conformational data obtained. The SAXS modeling revealed how significant propensity of Ubl conformations proximal to the USP domain distinctly shape the USP14 conformational ensemble (Figure 6). The presence of transient interactions between the Ubl and USP domains suggested from this ensemble is in full agreement with our NMR analysis, which reveals CSPs and dynamic exchange within and adjacent to the Ω_49–67_ loop in full‐length USP14 (Figure 3c), and restricted interdomain tumbling in the full‐length protein (Table 1) despite a highly flexible interdomain linker (Figure 4). The small size of the CSPs together with the dynamic modulations of intrinsic dynamics observed by NMR (Figures 3c and 4) suggest several transient interactions rather than a single mode of binding, which is in excellent agreement with the conformational ensemble derived from SAXS experiments. Our NMR and SAXS data are thus reconciled by a multiple states model that includes transient interactions between Ubl and USP domains and shapes the USP14 conformational ensemble.

### 2.6 The USP14 conformational ensemble is compatible with proteasome binding

To evaluate any possible functional relevance of the free USP14 conformational ensemble, we aligned it onto the USP domain of human USP14 in complex with the proteasome (Figure 6f). This recent cryo‐EM structure (Zhang et al., 2022) also includes Rpn1, to which USP14‐Ubl has been shown to bind (Shi et al., 2016). Interestingly, Ubl domains in Group 1 of the solution ensemble, which comprises the models with best fit to data (lowest *χ* ^2^), are in sufficient spatial proximity to easily approach the Rpn1 binding sites for the USP14‐Ubl domain (Shi et al., 2016; Zhang et al., 2022). Group 2 states are also accessible to the Ubl domain even within a USP domain–proteasome complex, with possible transient interaction to a helical region above the USP domain active site (Figure 6f). In Group 3 states, the Ubl positions would interfere with Rpn1 in a static complex but would be at correct distance and angle from the USP domain to facilitate binding to Rpn1 if only slightly shifted (Figure 6c,f). Taken together, a significant part of the USP14 structural ensemble is compatible with the proteasome‐bound USP14 structures observed by cryo‐EM (Zhang et al., 2022), suggesting that the free USP14 conformational ensemble may be biased toward conformations compatible with proteasome binding.

## 3 DISCUSSION

In this work, we have investigated the dynamic properties of the USP14‐Ubl domain in solution, both alone and in the context of the full‐length USP14 protein. The USP14 Ubl domain has been shown to be essential in activating the USP14‐USP enzyme by assisting its anchoring to the proteasome (Zhang et al., 2022) and may similarly be involved in other USP14 interactions (Doherty et al., 2022; Kuo and Goldberg, 2017; Ming et al., 2022; Srinivasan et al., 2019). Several USP‐containing DUBs contain Ubl domains that have been found to interact with USP domains and regulate their function by transient interactions at different time scales, as shown by NMR, SAXS, and/or kinetic analyses (Clerici et al., 2014; Faesen et al., 2011; Kim et al., 2019; Rougé et al., 2016). By joint interpretation of independent NMR and SAXS results as described above, we show that also in USP14, the Ubl and USP domains transiently interact, and that the free USP14 conformation is best described by a multiple states model. Furthermore, we find that this USP14 conformational ensemble is biased toward conformations compatible with proteasome binding already in the free state.

Our results indicate that USP14‐Ubl has similar backbone rigidity as ubiquitin but holds a different dynamic pattern in its loops. The USP14‐Ubl domain is structurally homologous to ubiquitin, a rigid protein that interacts with numerous proteins by collective and correlated intrinsic dynamics at distinct sites (Fenwick et al., 2011; Lange et al., 2008; Smith et al., 2016). In ubiquitin, its β1β2‐loop involved in protein interactions is highly dynamic whereas its β3β5‐loop, corresponding to the Ω_49–67_ loop in USP14, is less flexible (Chang and Tjandra, 2005; Lange et al., 2008) (Figure 4a). The reverse is true for USP14 where the β1β2‐loop dynamics do not deviate from average Ubl properties and the terminal ends of the Ω_49–67_ loop appear to be more flexible in USP14‐Ubl than in ubiquitin, at least within the ps‐ns timescale window investigated here (Lange et al., 2008). Interestingly, the presence of the USP domain dynamically affects residues K30 and D55, which in ubiquitin form a conserved salt bridge that stabilizes the loop corresponding to Ω_49–67_ in USP14 (Nandi and Ainavarapu, 2022; Walters et al., 2004). Residues displaying significant CSPs between free Ubl and USP14 are close in space to the Ω_49–67_, in agreement with USP influence on this part of the Ubl. Possibly, the Ubl domain of USP14 has evolved distinct flexible regions in a similar fashion as ubiquitin to allow for specific target binding while retaining core rigidity (Lange et al., 2008).

Our NMR analysis of the Ubl domain free and linked to the USP domain suggests that the dynamic Ω_49–67_ loop in USP14‐Ubl transiently interacts with the USP domain. Interestingly, recent cryo‐EM work suggests that the USP14 Ω_49–67_ loop is also involved in proteasome binding. Specifically, in the cryo‐EM structure of proteasome‐bound USP14, K45 is positioned between two α‐helices of Rpn1 (Zhang et al., 2022) (Figure 4a), suggesting a direct role in the USP14‐Rpn1 interaction. Furthermore, in complex with the 19S RP the USP14 Ω_49–67_ loop adopts slightly different conformations in open and closed gate states (PDB‐ID 7W3K, 7W3J [Zhang et al., 2022]), suggesting that the modulated dynamics that we observe for this loop in free USP14 may also have a functional role. Notably, we observe large *R*_2_ values for K45 and V46 already in the free state, with *R*_1_ *R*_2_ and *R*_2_/*R*_1_ values indicating chemical exchange, possibly between states corresponding to the free and proteasome‐bound (Figure 4f). Furthermore, a W58A mutation found to inhibit proteasome binding (Srinivasan et al., 2019) would also disrupt anchoring of Ω_49–67_ to the β2α1 interface (Figure 4a); such a mutation would dramatically affect both structure and dynamics in this part of the Ubl. Together with the high degree of inter‐species conservation of the USP14 Ω_49–67_ (Nandi and Ainavarapu, 2022; Srinivasan et al., 2019), this supports a functional role of this loop in inter‐ as well as intradomain interactions.

We find that in the conformation ensemble of free USP14, the Ubl domain is dynamically positioned adjacent to two regions of the USP domain: a loop including residues 385–416 (Group 1), and a set of α‐helices above the catalytic site (Group 2) (Figure 6c). Alignment of our USP14 ensemble onto the USP domain of USP14 in a cryo‐EM structure of the proteasome (Zhang et al., 2022) reveals that Group 1 Ubl positions are close to its Rpn1 binding site on the proteasome (Figure 6f). Interestingly, USP14 residues 385–416, which correspond to an unstructured loop in crystal structures of its USP domain alone (Hu et al., 2005; Wang et al., 2018), were recently shown by cryo‐EM to bind directly to the proteasome (Zhang et al., 2022) (Figure 6f). Transient Ubl interactions to the USP_385–416_ loop may therefore dynamically support favorable relative pre‐positioning of the Ubl and USP domains to interact with the proteasome. The Group 2 possible touch‐point region above the catalytic site in USP14 is a regulatory site in several DUBs: in USP7 it transiently interacts with Ubl domains in the same protein (Kim et al., 2019; Li et al., 2016; Rougé et al., 2016), and in the SAGA‐DUB complex, it binds the Sgf11 zinc‐finger domain to integrate Ubp8 (homolog of human USP22) as an active DUB (Samara et al., 2012). In the absence of proteasome, the presence of the Ubl domain did not give any significant inhibitory nor activating effects on ubiquitin‐rhodamine hydrolysis, which is an assay commonly used to measure DUB activity. Full USP14 activity may require allosteric nudging at several sites on its USP domain initiating both global and local enzyme changes, as shown for USP12 and USP1 (Li et al., 2016; Rennie et al., 2021). The low atomic and time resolution of the free USP14 ensemble shown here, does not allow for detailed structural interpretations of how transient interdomain interactions would enhance proteasome binding. However, a transient interdomain interaction within free USP14 that would bias its conformational space toward the bound state would reduce the entropic cost of binding, which in turn may increase proteasome affinity and thereby also increase USP14 activity which is turned on by proteasome binding. In this context, it is interesting that the proteasome affinity to intact USP14 was recently found to be higher than to either of its subdomains (Zhang et al., 2022). The dynamically bipartite nature of the USP14 linker region that we observe in solution could also support proteasome binding, where the more rigid part could facilitate appropriate distancing between the two domains and the highly flexible linker region closest to the Ubl would facilitate proper sampling of states suitable for binding of the Ubl and USP domain to the proteasome.

Taken together, our results propose a multiple states model for free USP14. In this model, USP14 binding to the proteasome could be facilitated by transient interdomain interactions and linker‐biased conformational sampling, leading to USP14 conformational ensembles predisposed toward the proteasome‐bound state already in the absence of proteasome. This ensemble view of free USP14 extends the current perspective, where the USP14‐Ubl domain has primarily been considered as a binding anchor for USP14 binding to the proteasome (Hu et al., 2005; Zhang et al., 2022). Further evaluation of full‐length USP14 conformational dynamics in the absence and presence of proteasome will be required to understand these properties in full.

## 4 METHODS

### 4.1 Protein production and purification

Non‐isotopically 6xHis‐tagged human USP14 and its two domains were produced as previously described (Selvaraju et al., 2019). ^13^C‐ and ^15^N‐labeled USP14_1–80_ and USP14_1–494_ was cloned into pNIC‐28‐Bsa4 vector and expressed in *E. coli* BL21(DE3) Ros2 cells and grown in M9 minimal medium enriched with ^13^C‐D‐glucose and ^15^NH_4_Cl, at 37°C until OD_600_ = 0.6. The culture was induced with 0.5 mM IPTG and harvested after 18 h at 18°C. The pellet was resuspended in 20 mM HEPES pH 7.5, 500 mM NaCl, 10 mM imidazole, 5% glycerol, 0.5 mM TCEP, 5 units/mL recombinant DNAse I and 1 EDTA‐free protease inhibitor cocktail tablet per 75 mL, and lysed by sonication using a Branson Digital Sonifier 250 for 3 min with the parameters 10 s on, 10 s off, and 30% amplitude. The purification followed the same procedure as non‐isotopically labeled USP14.

### 4.2 NMR spectroscopy

Data were collected on Varian INOVA 600 MHz NMR spectrometer equipped with a cryoprobe. USP14_1–80_ and USP14_1–494_ were concentrated to 0.4 and 0.3 mM, respectively, in 20 mM HEPES pH 6.9, 100 mM NaCl, 0.5 mM TCEP, 0.02 mM NaN_3_, and 10% D_2_O. All spectra were processed with NMRpipe (Delaglio et al., 1995) or mddNMR (Qu et al., 2015). Experiments for backbone resonance assignment included HSQC, HNCO, HNcaCO, HNCACB, CBCAcoNH, and additionally HNCA for USP14_1–494_, and were assigned using NMRFAM‐SPARKY (Lee et al., 2015) aided by COMPASS (Niklasson et al., 2015). All experiments were performed at 25°C except for backbone resonance assignment of USP14_1–494_, which were performed at 30°C for increased sensitivity. CSPs were calculated using HSQC spectra of USP14_1–80_ and USP14_1–494_, at 25°C and using the formula:

$$\Delta \delta =\sqrt{{\Delta \delta }_{\mathrm{HN}}^{2}+{\left(\frac{{\Delta \delta }_{N}}{R_{\text{scale}}}\right)}^{2},}$$

with the scaling factor *R*_scale_ = 6.5 (Mulder et al., 1999). The trimmed mean CSP and corresponding standard deviation (*σ*) as shown in Figure 3 were calculated stepwise by first calculating the mean and *σ* of the whole data set, after which any value exceeding one *σ* was excluded and a new mean and *σ* was calculated for the remaining values; this was done in two iterations due to the large peak movement of E80 and the last calculated mean and *σ* was used for data interpretation. Secondary structure populations were estimated from N, H_N_, C_α_, C_β_, and C′ chemical shifts using CheSPI for data collected in this study and for a previous solved NMR structure of the Ubl domain for the USP14 mouse homolog (PDB‐ID 1WGG, BMRB 11256) which was measured in 20 mM PiNa, 100 mM NaCl, 1 mM d‐DTT, 0.02% NaN_3_, 10% D_2_O, pH 6.0, 24.85°C at 800 MHz (Zhao et al., 2004) (Figure S1).

### 4.3 NMR relaxation measurements

^15^N *R*_1_ and *R*_1ρ_ relaxation, hetNOE, and CPMG relaxation dispersion experiments were collected as pseudo‐3D for the same samples described above. ^15^N *R*_1_ relaxation were collected at 14 or 15 different delays and 4 or 3 duplicates: 10.1, 10.1, 20.2, 20.2, 40.3, 40.3, 80.6, 80.6, 120.9, 161.2, 201.5, 302.3, 352.7, 403.1, 453.5, 503.8, 584.5, 655 ms for USP14_1–494_ and 10.1, 10.1, 20.2, 40.3, 80.7, 80.7, 121, 161.3, 201.6, 252, 302.4, 352.9, 403.3, 453.7, 504.1, 554.5, 645.2, 645.2 ms for USP14_1–80_. *R*_1ρ_ were collected at 15 or 17 unique delays and 3 duplicates: 6, 6, 9, 12, 15, 18, 24, 30, 40, 50, 60, 60, 70, 85, 100, 100, 115, 130 ms for USP14_1–494_ and 6, 6, 12, 15, 18, 24, 30, 36, 42, 50, 60, 70, 80, 90, 90, 100, 110, 120, 130, 130 ms for USP14_1–80_. HetNOE experiments were recorded in duplicates with and without 5 s ^1^H saturation. ^15^N CPMG relaxation dispersion (Hansen et al., 2008) were collected with a constant‐time relaxation delay of 30 ms and frequencies ranging between 33 and 1000 Hz. Relaxation rates and errors were determined using the software PINT, and the same software was used for calculating *R*_2_ relaxation rate constants from *R*_1_ and *R*_1ρ_ (Ahlner et al., 2013). Peaks were integrated and fitted with Gaussian line shapes for the CPMG experiments, and a combination of Gaussian and Lorentzian line shapes for the *R*_1_, *R*_2_, and hetNOE experiments. Errors were estimated by the jackknife approach. Overlapping residues were excluded from the analysis. Mean calculations for relaxation experiments were done as described above for one iteration. TENSOR2 was used for calculations of the rotational correlation time (*τ*_c_) and order parameters (*S* ^2^) using *R*_1_, *R*_2_, and hetNOE values (Dosset et al., 2000). *τ*_c_ was calculated for residues with hetNOE values higher than 0.65. Theoretical *τ*_c_ was estimated using HYDRONMR 7.0c (de la Torre et al., 2000) using the recommended AER of 3.3 Å with AlphaFold model of USP14‐Ubl (residue 1–75) and USP14‐USP crystal structure in complex with ubiquitin (PDB‐ID 2AYO).

### 4.4 DUB activity assay

Samples of 5 μM USP14_1–494_ or USP14_99–494_ were prepared in triplicates in a black 384‐well plate (Corning 3820) with reaction volume of 20 μL. The assay buffer contained 50 mM HEPES pH 7.5, 50 mM NaCl, 5 mM MgCl_2_, 1 mg/mL BSA, 1 mM DTT, and 2 mM ATP; 1 μM ubiquitin‐rhodamine (Bio‐Techne, U‐555‐050) was added to start the reaction. The hydrolysis of ubiquitin‐rhodamine was monitored with a Promega GloMax plate reader at 475 nm at 37°C. The average background was subtracted, and the reaction rate was calculated by simple linear regression and significance was evaluated by *t*‐test in Graphpad Prism 10.

### 4.5 Small angle x‐ray scattering measurements

SAXS data were collected at EMBL PETRA‐III P12 beamline (DESY, Hamburg, Germany) (Blanchet et al., 2015) at room temperature using SEC‐SAXS for USP14_1–494_ and USP14‐_91–494_ as described in Table 2. Data was processed with CHROMIXS (Panjkovich and Svergun, 2018), and the scattering profile was analyzed using PRIMUS and GNOME within the ATSAS package (Konarev et al., 2003; Manalastas‐Cantos et al., 2021; Svergun, 1992). Fit of models or structures to experimental data was done with CRYSOL (Svergun et al., 1995). Bound ligands and waters were removed prior to fit. Chain A was used for structures containing several chains.

### 4.6 Modeling of the USP14 solution structural ensemble

To first map the theoretically accessible space of USP14 interdomain positions, an initial set of 13,000 USP14 interdomain conformations were generated by sampling different conformations of the loop connecting the two domains in USP14. No experimental restraints were used in this stage. A starting model of USP14 was modeled using AlphaFold2 (Jumper et al., 2021), and the model was energy minimized in Rosetta's force field using the relax application with the option *relax:constrain_relax_to_start_coords* enabled. Starting from the relaxed AlphaFold model of USP14 (*AF.rlx.pdb* in Rosetta command line below), the relative positioning of the two USP14 domains was explored by sampling the backbone degrees of freedom of the loop residues (76–102 residue position) connecting the two domains using the FloppyTail application in Rosetta (Kleiger et al., 2009). The backbone degrees of freedom were sampled with 3‐mer backbone fragments and smaller changes to individual backbone psi/phi dihedrals. The backbone fragments were taken from known structures with local sequences similarity and secondary structure preference. In total, 13,000 conformations were generated using Monte Carlo energy minimization by first sampling the backbone orientations in low‐resolution followed by all‐atom refinement. The Rosetta command line used to generate the 13,000 conformations is shown below:


FloppyTail.mpi.linuxgccrelease


‐in:file:s *AF.rlx.pdb*

‐nstruct 13000


‐out:file:silent silent.out


‐packing:repack_only


‐in:file:movemap *movemap.dat*

‐FloppyTail::perturb_cycles 10000


‐FloppyTail::refine_cycles 1000


‐in:file:frag3 frags.200.3mers


‐FloppyTail:C_root.





The *movemap.dat* file instructs the protocol to sample the backbone and side‐chains degrees of freedom for residue 76–102:


RESIDUE * NO


JUMP * NO


RESIDUE 76102 BBCHI





The SAXS‐guided molecular USP14 solution structural ensemble was constructed from the initial 13,000‐membered structural space using the iBME (Pesce and Lindorff‐Larsen, 2021). In short, SAXS profiles were calculated from each structure in the initial 13,000‐membered ensemble, and iBME was then used to obtain normalized weights (*w*_*i*_) representing their contribution to a *χ* ^2^‐optimized iBME fit of an ensemble of SAXS profiles to the experimental USP14 SAXS data. The final USP14 solution structural ensemble was defined as the structures corresponding to the SAXS profiles that contributed 0.5% or more (*w*_*i*_ > 0.005) to the iBME fit, resulting in an ensemble of 30 structures.

### 4.7 Accession numbers

Chemical shift assignments and NMR relaxation parameters are deposited in BMRB with accession numbers 51684 (USP14_1–80_) and 51685 (USP14_1–494_). SAXS data is deposited in SASBDB with accession numbers SASDQZ7 (USP14_1–494_) and SASDQ28 (USP14_91–494_). The SAXS‐based conformational ensemble for USP14_1–494_ is included in SASDQZ7.

## AUTHOR CONTRIBUTIONS

**Alexandra Ahlner:** Conceptualization; investigation; writing – original draft; writing – review and editing; supervision; project administration. **Johannes Salomonsson:** Conceptualization; methodology; investigation; writing – original draft; writing – review and editing; formal analysis; visualization. **Bjorn Wallner:** Investigation; writing – review and editing; writing – original draft; methodology; software; formal analysis; funding acquisition. **Linda Sjöstrand:** Investigation; formal analysis; writing – review and editing. **Pádraig D'Arcy:** Supervision; writing – review and editing; funding acquisition. **Maria Sunnerhagen:** Conceptualization; writing – original draft; writing – review and editing; supervision; funding acquisition; project administration.

## CONFLICT OF INTEREST STATEMENT

The authors declare no conflicts of interest.

## Supporting information


**Figure S1.** Summary of CheSPI evaluations based on chemical shift data from hUSP14_1–494_, hUSP14_1–80_, and mUSP14_4–86_. (A) Weighted difference between observed and predicted shifts shown with blue, red, black, cyan, and magenta dots for C′, C_α_, C_β_, H_N_, and N, respectively. (B) CheSPI components (blue and red) and CheZOD *Z*‐scores (black). Green dashed lines at *Z* = 8.0 for reference, and CheZOD *Z*‐scores <8 are classified as disordered. (C) Bar plot colored according to the CheSPI color scheme. CheZOD *Z*‐scores are used for bar heights. (D) Secondary structure populations as shown in Figure 2b. (E) Illustration of the most confident secondary structure prediction.

**Figure S2.** DUB activity assay for USP14_1–494_ (purple) and USP14_99–494_ (blue). (A) Representative graph of ubiquitin‐rhodamine (ub‐rho) hydrolysis. (B) Ub‐rho hydrolysis rates from four separate experiments.

**Figure S3.** SAXS data of USP14_1–494_ (purple) and USP14_91–494_ (blue). (A) Scattering curve, (B) Guinier plot and linear fit, (C) pair‐wise distance distribution, and (D) dimensionless Kratky plot. Dotted lines in the Kratky plot indicate the maximum point of a typical globular shape.

**Figure S4.** Theoretical *R*_g_ values of generated models. (A) Distribution of the number of models as a function of *R*_g_ for all generated models in the initial set of models (blue) and in the iBME‐selected SAXS‐based conformational ensemble (orange). The higher number of models with low *R*_g_ in the initial set of 13,000 models is consistent with the known bias of Rosetta‐Monte Carlo protocols toward the generation of more compact models. (B) The fit of models to SAXS‐data as a function of *R*_g_ for the individual models in the conformational ensemble. Models in A and B correspond to Groups 1–3 in Figure 6 as indicated on top of Figure S4A.

**Table S1.** Fits of USP14‐USP structures and models to SAXS data of USP14_91–494_ using CRYSOL. The AlphaFold model used was AF‐P54578‐F1.
