# Supplementary figures and images for: Transient interdomain interactions in free USP14 shape its conformational ensemble
Source: Protein Sci. 2024 Apr 8;33(5):e4975. doi: 10.1002/pro.4975 (PMC11001199; doi:10.1002/pro.4975)

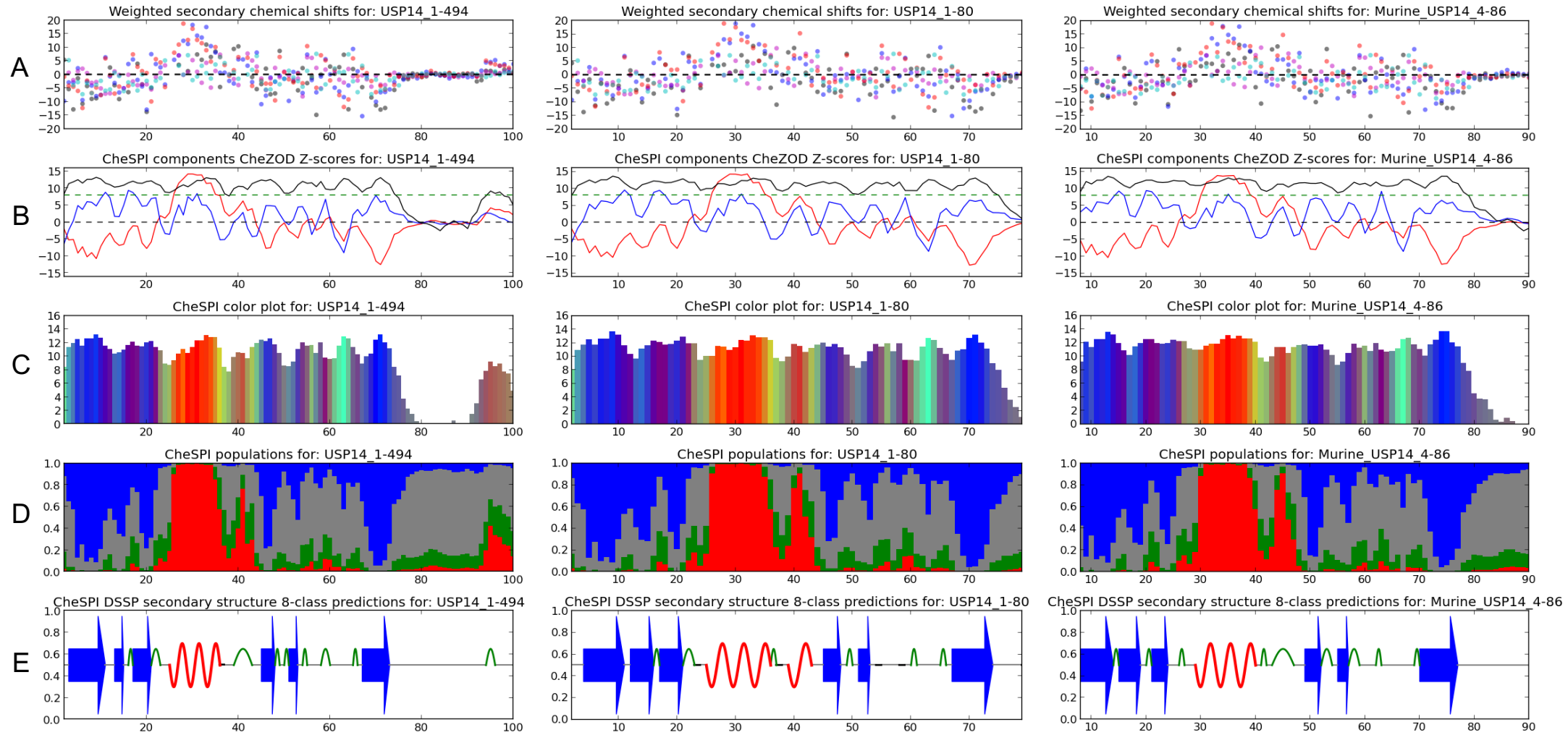

Supplement: Supplementary file 1 — Figure S1. Summary of CheSPI evaluations based on chemical shift data from hUSP141–494, hUSP141–80, and mUSP144–86. (A) Weighted difference between observed and predicted shifts shown with blue, red, black, cyan, and magenta dots for C′, Cα, Cβ, HN, and N, respectively. (B) CheSPI components (blue and red) and CheZOD Z‐scores (black). Green dashed lines at Z = 8.0 for reference, and CheZOD Z‐scores <8 are classified as disordered. (C) Bar plot colored according to the CheSPI color scheme. CheZOD Z‐scores are used for bar heights. (D) Secondary structure populations as shown in Figure 2b. (E) Illustration of the most confident secondary structure prediction. [file PRO-33-e4975-s004.pdf]

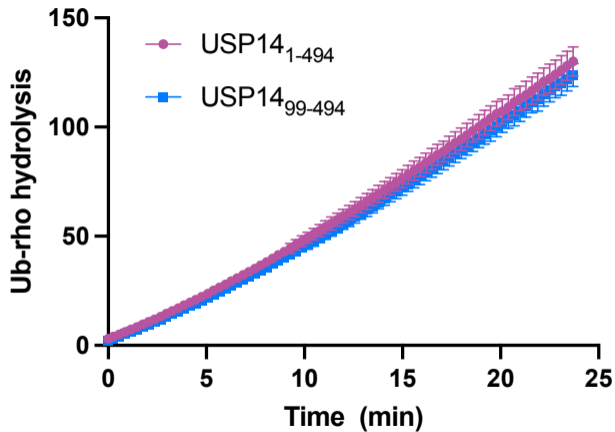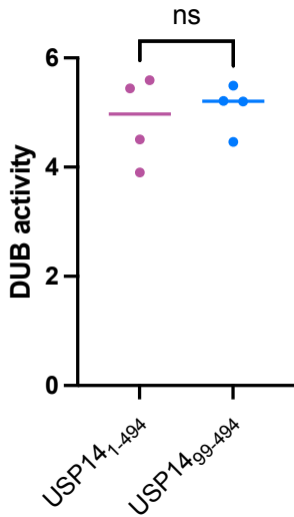

Supplement: Supplementary file 2 — Figure S2. DUB activity assay for USP141–494 (purple) and USP1499–494 (blue). (A) Representative graph of ubiquitin‐rhodamine (ub‐rho) hydrolysis. (B) Ub‐rho hydrolysis rates from four separate experiments. [file PRO-33-e4975-s003.pdf]

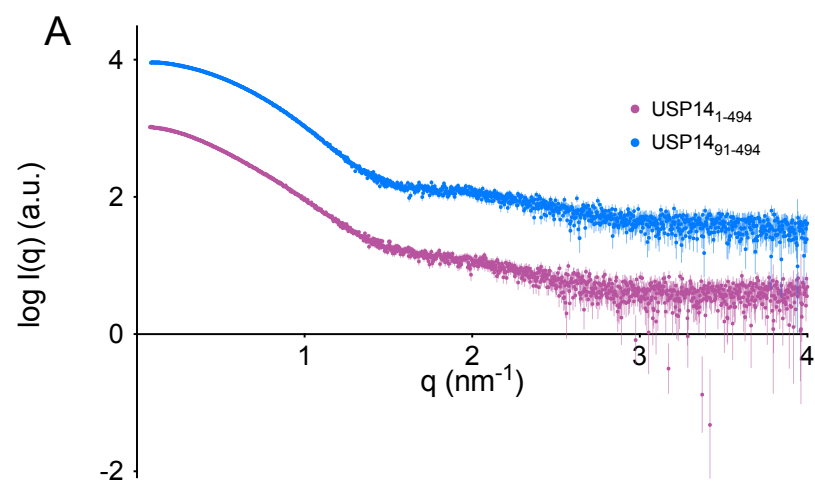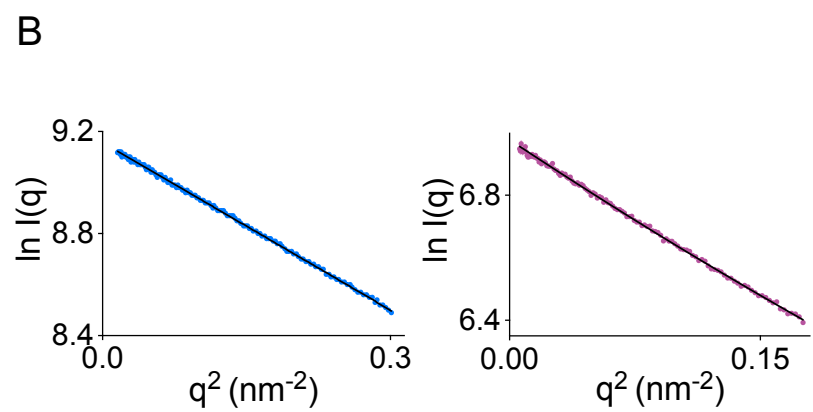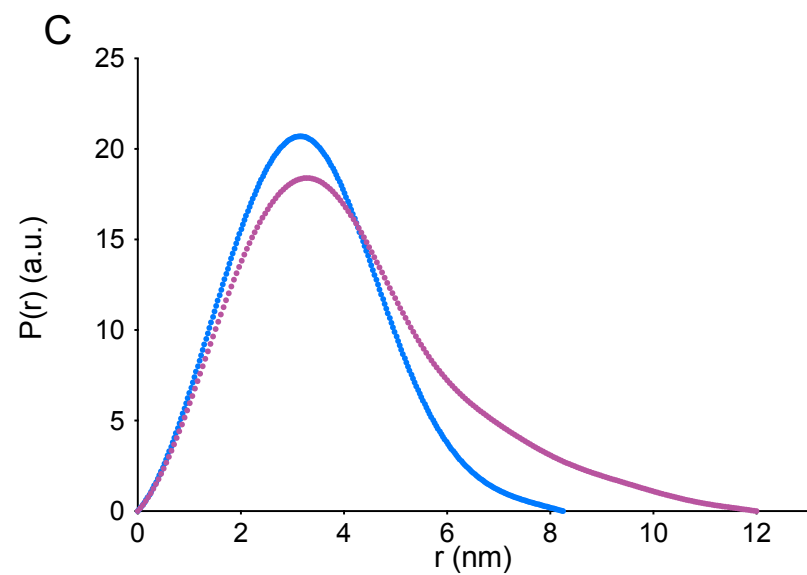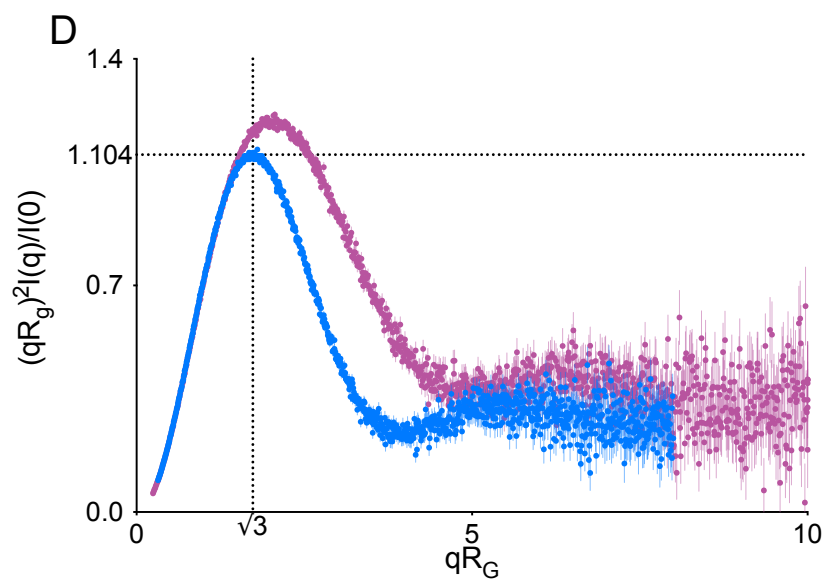

Supplement: Supplementary file 3 — Figure S3. SAXS data of USP141–494 (purple) and USP1491–494 (blue). (A) Scattering curve, (B) Guinier plot and linear fit, (C) pair‐wise distance distribution, and (D) dimensionless Kratky plot. Dotted lines in the Kratky plot indicate the maximum point of a typical globular shape. [file PRO-33-e4975-s005.pdf]

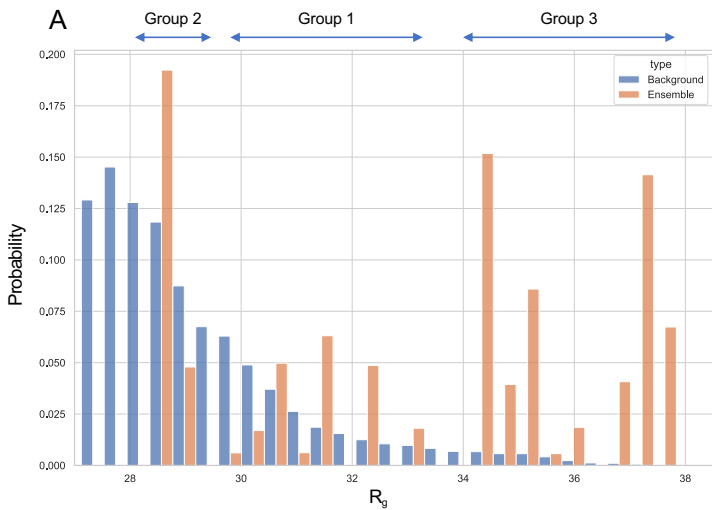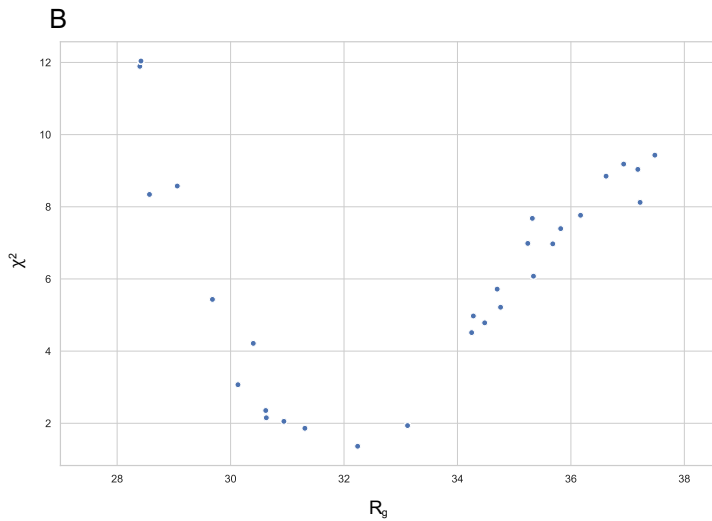

Supplement: Supplementary file 4 — Figure S4. Theoretical R g values of generated models. (A) Distribution of the number of models as a function of R g for all generated models in the initial set of models (blue) and in the iBME‐selected SAXS‐based conformational ensemble (orange). The higher number of models with low R g in the initial set of 13,000 models is consistent with the known bias of Rosetta‐Monte Carlo protocols toward the generation of more compact models. (B) The fit of models to SAXS‐data as a function of R g for the individual models in the conformational ensemble. Models in A and B correspond to Groups 1–3 in Figure 6 as indicated on top of Figure S4A. [file PRO-33-e4975-s002.pdf]
