# Supplementary material for: Transient interdomain interactions in free USP14 shape its conformational ensemble
Source: Protein Sci. 2024 Apr 8;33(5):e4975. doi: 10.1002/pro.4975 (PMC11001199; doi:10.1002/pro.4975)
Supplement: Supplementary file 5 — Table S1. Fits of USP14‐USP structures and models to SAXS data of USP1491–494 using CRYSOL. The AlphaFold model used was AF‐P54578‐F1. [file PRO-33-e4975-s001.pdf]

Structure/model	Sequence length	R_g	χ^2	RMSD to AlphaFold
2AYN	100-482	2.48	2.71	0.390
2AYO	97-482	2.49	2.19	0.559
6IIK	102-485	2.39	14.47	0.361
6IIL	102-487	2.45	4.49	0.323
6IIM	102-485	2.42	9.13	0.354
6IIN	101-485	2.38	17.92	0.305
6LVS	103-484	2.38	17.24	0.391
I-TASSER model	91-494	2.47	4.26	1.110
AlphaFold model	91-494	2.53	1.06	N/A
